# Diet breadth modulates preference - performance relationships in a phytophagous insect community

**DOI:** 10.1038/s41598-017-17231-2

**Published:** 2017-12-05

**Authors:** Maud Charlery de la Masselière, Benoît Facon, Abir Hafsi, Pierre-François Duyck

**Affiliations:** 1 CIRAD, UMR PVBMT, 97410 Saint Pierre, La Réunion France; 2 Université de la Réunion, 97400 Saint Denis, La Réunion France; 3 INRA, UMR PVBMT, 97410 Saint Pierre, La Réunion France; 4 Institut supérieur agronomique de Chott-Mariem, Laboratoire d’entomologie et de lutte biologique, Université de Sousse, 4042 Sousse, Tunisia

## Abstract

In most phytophagous insects, larvae are less mobile than adults and their fitness depends on the plant chosen by their mother. To maximize fitness, adult preference and larval performance should thus be correlated. This correlation is not always apparent and seems to increase with the level of specialisation, i.e. specialists have a stronger preference for high quality host plant species compared to generalists. The aim of this study was to test whether the relationship between female preference and larval performance was stronger for specialists than for generalists within a community of fruit flies (Diptera: Tephritidae). A total of six fruit fly species was used, including four generalists, and two specialists co-existing in La Reunion island (France). We estimated oviposition preference through the number of eggs laid and larval performance through the larval survival on 29 different host plants species belonging to 15 families in the laboratory and evaluated the relationship between these two traits. Preference-performance relationship differed according to the degree of specialisation with a strong positive correlation for specialists and no relationship for generalists. These results substantiate the theory that choosing high quality hosts is more important for specialists that are adapted to survive on fewer host plants than for generalists.

## Introduction

Phytophagous insects comprise a substantial proportion of all described macro-organisms on earth with around five million described species^1^. Most phytophagous species are specialised on only a few plant species for feeding during development, while others can feed and develop on a multitude of host plants. In order to make better predictions of plant-insect interactions in new environments, it is crucial to evaluate differences between specialists and generalists in their feeding strategies by studying both adult preference (i.e. the non-random choice of hosts for oviposition^2,3^) and larval performance (i.e. the capacity of larvae to develop and survive on the host)^2–8^. In most phytophagous insects, larvae are less mobile than adults; hence the success of larval development depends on the quality of the plant chosen by the adult^2^. By choosing among resources that differ in nutritional quality for larvae, adult oviposition behaviour is expected to be under strong selection to maximize fitness of the offspring^3^. Responses to such selection pressures are expected to lead to a positive correlation between behavioural traits involved in adult preference and physiological traits involved in larval performance, also known as the *“mother knows best hypothesis*”^2,4,9^.

Whereas some studies on phytophagous insects show a clear positive relationship between adult preference and larval performance^10–13^, others do not^14–16^. A meta-analysis suggested that the mismatch between preference and performance traits could be due to different degrees of specialisation^4^. Specialists (i.e. species feeding on plants belonging to a single family) tend to show a stronger relationship between female preference and larval performance than generalists (i.e. species feeding on plants belonging to several families), because specialists have a higher fitness on a restricted host range^17^. In contrast, generalists are able to survive on many hosts without being optimally adapted to any plants due to the cost of adaptation^18^ (with the exception of some species^19^). The choice of a low quality plant will thus have less severe consequences for generalists compared to specialists. Recent empirical studies comparing one generalist and one specialist within the same ecosystem provide additional support that generalists fare better on low quality hosts compared to specialists^20,21^. Similar findings were obtained when a higher number of related species was analysed across different ecosystems^22,23^. Despite these studies, interpreting preference-performance relationships is challenging, because often a limited number of host plants are used in comparisons. More importantly, differences between specialists and generalists using a single species *per* degree of specialisation (i.e. specialist or generalist) and within different environments can either be due to taxon-specific responses or to adaptations within a common environment rather than degree of specialisation^24,25^.

We focused on the relationship between female preference and larval performance of six species of true fruit flies (Diptera: Tephritidae) belonging to the same community on the island La Réunion. Within this community, two species, *Dacus demmerezi* and *Zeugodacus cucurbitae* (formerly known as *Bactrocera cucurbitae* [De Meyer, *et al*.^26^]), feed mostly on the cucumber family (Cucurbitaceae)^27–29^ and four species, *Ceratitis catoirii*, *C*. *capitata*, *C*. *quilicii* (formerly known as *Ceratitis rosa* [De Meyer, *et al*.^26^]) and *Bactrocera zonata*, feed on an average of 36 plants (min: 16, max: 60) belonging to 17 different^27,30,31^. This community includes two native species (*C*. *catoirii* and *D*. *demmerezi*) and the other species successively invaded the island from Asia (*Z*. *cucurbitae* in 1972 and *B*. *zonata* in 2000) or Africa (*C*. *capitata* in 1939, *C*. *quilicii* in 1955). Abundance and distribution of tephritids in this community are also influenced by interspecific competition^32,33^, leading to niche partitioning through climatic and host plant differences^31,34^. The aim of our study was to test whether the relationship between female preference and larval performance was stronger for specialists than for generalists within the Tephritidae community in La Réunion. To test this relationship, we evaluated female preference by the oviposition through a choice and a no-choice experiment and larval performance through the survival of each Tephritidae species on fruits from 29 plants species belonging to 15 families (Table 1). We then compared our results between generalist and specialist species that share a common environment and take into account the degree of specialisation.

**Table 1:** Fruit species tested to study the preference – performance relationship in six Tephritidae species. *Indicates fruits used in the choice experiment.

Family	Scientific name	Common name	ID
Anacardiaceae	Mangifera indica*	Mango	1
Annonaceae	*Annona reticulata*	Custard apple	2
Cactaceae	*Hylocereus undatus*	Dragon fruit	3
Caricaceae	*Carica papaya*	Papaya	4
Combretaceae	Terminalia catappa*	Indian almond	5
Cucurbitaceae	*Citrullus lanatus*	Watermelon	6
	Cucumis melo*	Melon	7
	*Cucumis sativus*	Cucumber	8
	Cucurbita maxima*	Pumpkin	9
	*Cucurbita pepo*	Zucchini	10
	*Sechium edule*	Chayote	11
Lauraceae	*Persea Americana*	Avocado	12
Lythraceae	*Punica granatum*	Pomegranate	13
Moraceae	*Ficus carica*	Fig	14
Myrtaceae	Psidium cattleyanum*	Strawberry guava	15
	Psidium guajava*	Guava	16
	*Syzygium jambos*	Rose apple	17
	*Syzygium samarangense*	Java apple	18
Oxalidaceae	*Averrhoa carambola*	Star fruit	19
Rosaceae	*Eriobotrya japonica*	Loquat	20
	*Prunus domestica*	Plum	21
	*Prunus persica*	Peach	22
Rubiaceae	*Coffea arabica*	Coffee	23
Rutaceae	*Citrus reticulata x Citrus sinensis*	Tangor	24
Solanaceae	Capsicum annum*	Chili	25
	*Solanum betaceum*	Tree tomato	26
	Solanum lycopersicum*	Tomato	27
	*Solanum mauritianum*	Bugweed	28
	*Solanum melongena*	Eggplant	29

## Results

### Classification into generalists and specialists

Generalist species had significantly higher alpha diversity of preference (based on the number of eggs laid) and performance (based on survival probability) on host plants than specialist species. Specifically, generalists had a higher alpha diversity on hosts than specialists, justifying our classification into these two groups (Supplementary Fig. S1).

### Adult preference

**Figure 1:**
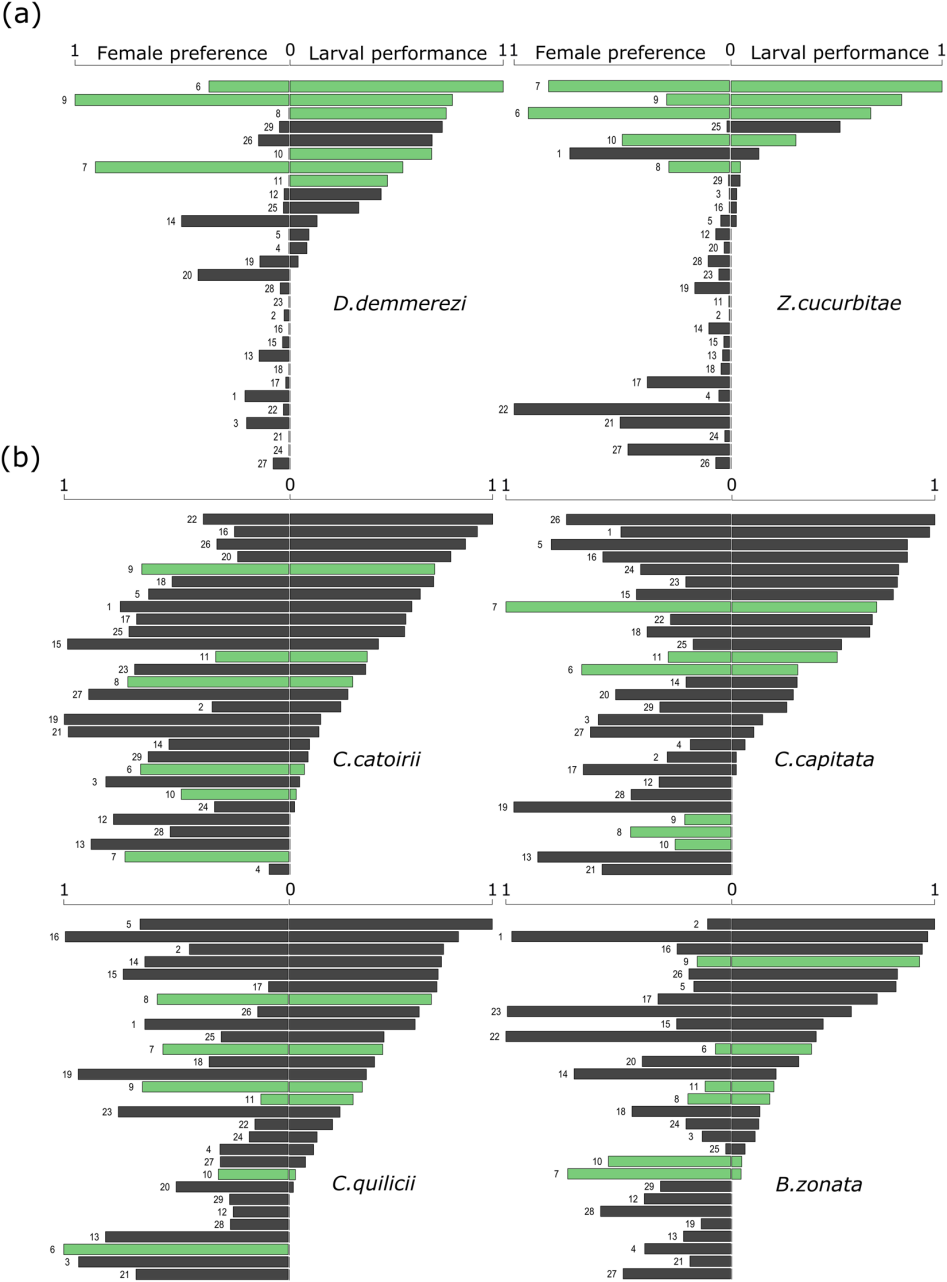
Female preference and larval performance for each (**a**) specialist species: *D*. *demmerezi* and *Z*. *cucurbitae* and each (**b**) generalist species: *C*. *catoirii*, *C*. *capitata*, *C*. *quilicii* and *B*. *zonata*. Each number represents one plant species and the correspondence between the name and number can be found in the Table 1. Green bars correspond to fruits belonging to the Cucurbitaceae family.

The oviposition rate was strongly influenced by fruit species (Δ*dev*_28, 1068_ = 8773.4, p-value < 0.0001) and the interaction with degree of specialisation, expressed by the alpha diversity value (Δ*dev*_28, 1039_ = 8238.7, p-value < 0.0001). Specialist species mostly preferred plants belonging to the Cucurbitaceae family, such as melon, pumpkin and watermelon (Fig. 1).

### Larval performance and its relationship with adult preference

**Figure 2:**
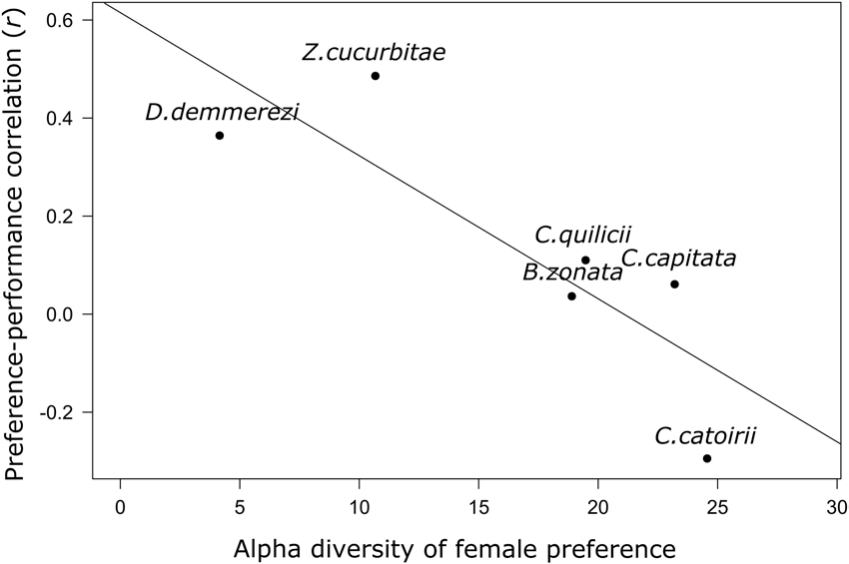
Relationship between the strength of the preference – performance relationship and the diet breadth of the Tephritidae species. For each Tephritidae species, the correlation between the female preference and the larval performance is assessed by the Pearson correlation index *r*, and the diet breadth is assessed by mean number equivalent alpha diversity of the female preference.

**Figure 3:**
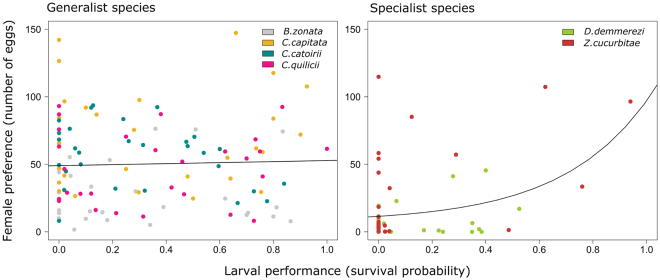
Relationship between adult preference and larval performance for generalist and specialist species. The female preference is represented by the number of eggs laid. Within each degree of specialisation, species are represented by a distinct colour.

Larvae of specialist species had a higher survival on few plants belonging to the Cucurbitaceae family compared to generalist species that survived on plants of different families (Fig. 1, for more details see^35^). There was a significant positive correlation (*r* = 0.82, p-value = 0.038) between the alpha diversity values calculated using number of eggs laid in the no-choice setting and larval survival (Supplementary Fig. S1), which is in line with the finding that specialists choose and survived on fewer host plants than more generalist species. The relationship between adult preference and larval performance differed significantly between generalist and specialist species (Δ*dev*_1,170_ = 287.14, p-value = 0.0001) (Fig. 2). Within generalists, there was no significant effect of larval survival on adult preference (Δ*dev*_1,114_ = 1.94, p-value = 0.719). In contrast, within specialists there was a significant effect of larval survival on oviposition preference of females (Δ*dev*_1, 56_ = 335.53, p-value = 0.0012) (Fig. 3). To confirm that the significance of the result is not due to the presence of many zeroes, we performed analyses to test if specialist species selected the best host for larvae when they lay eggs. The effect of the larval survival on the oviposition of the females for specialist species was significantly positive when removing the zeroes in the larval survival (Δ*dev*_1,23_ = 257.46, p-value = 0.008), the female oviposition (Δ*dev*_1,46_ = 298.99, p-value = 0.003) and both (Δ*dev*_1,18_ = 196.24, p-value = 0.02).

There were no significant differences among species in the relationship between oviposition and larval survival within generalists (Δ*dev*_3,108_ = 30.72, p-value = 0.563) and within specialists (Δ*dev*_1,54_ = 14.58, p-value = 0.479).

## Discussion

Our study on the Tephritidae community of La Réunion showed a positive relationship between adult preference and larval performance for specialist species, but no such relationship was found for generalist species. We confirmed results of a meta-analysis performed by Gripenberg, *et al*.^4^, but here only species were used with the same degree of specialisation and from the same environment. This study is, to our knowledge, the first empirical work comparing the impact of degree of specialisation on the correlation between preference and performance within a phytophagous insect community. Previous studies using species from different communities showed contradictory results concerning the role of degree of specialisation on the relationship between preference and performance^20,23^. While a study on bollworms (Lepidoptera: Noctuidae) showed a stronger relationship between preference and performance for specialist species^20^, another study on butterflies (Lepidoptera: Nymphalidae) showed that the difference in the relationship between preference and performance was due to the clutch size instead of the degree of specialisation^23^. Our study, however, confirmed the theory that generalists lack a strong relationship between preference and performance^4,36^.

Few studies have investigated the preference-performance relationship for tephritids and so far no study has found a relationship between preference and performance for the generalist species *Ceratitis capitata* ^37,38^. It was shown, however, that specialised species in the genus *Dacus* preferred suitable hosts for larval development^39^. Clarke^40^ already highlighted that hosts of generalist species (with the example of the genus *Bactrocera*) are all ripened fruits with high sugar contents. This trait is well-conserved across fruit families, and does, therefore, not require a high level of discrimination, which could explain the mismatch found in some generalist tephritids. The nutritional composition of host fruits was recently shown to play a role in the larval performance of Tephritidae species in La Réunion with specialists performing better on fruits containing a high concentration of water and thus less sugar and generalists performing better on fruits containing higher concentrations of carbohydrate, fibre, and lipid^35^. In our study, specialist species were choosier than generalists. Specialisation enables females to make more precise decisions due to the restricted range of host plants they can successfully develop on^41^. This is an advantage for the development of their larvae as well as for minimizing searching time and avoiding predators^41^. Specialist females laid eggs on few hosts mostly belonging to the Cucurbitaceae family; hence many plants from other families were not preferred, including some plants on which no eggs were laid altogether. Specialist species are more adapted to their host but this adaptation may generate a trade-off between host suitability and host plant number leading to a restricted range of hosts^42^. In that case, females choosing the most suitable hosts for larval development are optimizing their fitness leading to an optimal offspring survival. As expected, generalist females laid eggs on almost all fruit species. A positive relationship between adult choice and larval performance was shown to be rule rather than exception^4^, but being selective has a cost in terms of energy and time^43,44^. The high variation in resource quality that generalists have to deal with may require more information to be integrated regarding the quality of their hosts^45^. This is potentially costly and makes them less choosy than specialists^36,46^. This lack of choosiness may explain the non-correlation between the choice and the no-choice experiment for *C*. *catoirii* and *C*. *quilicii*, because if they lay eggs on all plant species, the order of preference may change over the experiment. Moreover, larvae of generalists can survive in many fruits belonging to many families. The survival on many hosts may not be optimal because of the trade-off between host number and host quality. Despite this trade-off, generalism may not come at a high cost in some cases, as in mites^47^ where adaptation to a new host did not reduce performance and adaptation to the ancestral host. In our community, larvae of generalist tephritids are able to survive on many plants due to the very nutritive compounds of their hosts (i.e. a high sugar content and lack of toxic compounds)^35,40^. In such a situation, adults may not benefit from spending time to find and oviposit on a particular subset of host plants, since larvae can survive on many plants. When generalists select hosts of sub-optimal quality for larval development, the consequences will thus be less severe for generalists than specialists. The potential costs in time and energy of choosing particular hosts imply trade-offs between host selection and life-history traits such as fecundity. For example, insects with low fecundity should be more selective because they cannot afford losing some offspring^48^. Future studies could evaluate the costs experienced during host choice by generalists and specialists.

One limitation of our study is that species were reared on different diets. This was necessary to maintain the rearing, but could lead to different maternal effects that could influence the preference of females as well as performance of larvae. Specialist species were, however, reared on zucchini and our results showed that zucchini was not the preferred plant (fifth position for *Z*. *cucurbitae* and not selected at all for *D*. *demmerezi*) which suggests that the influence of the rearing diet on host choice was weak. We further measured performance only as larval survival, without analysing developmental time and pupal weight. These three traits were, however, all included in a recent study on Tephritidae species within the same community, and were shown to be correlated^35^, allowing us to hypothesize that survival can be used as a proxy of the larval performance.

The strength of our experimental design is that it allows a comparison of six Tephritidae species on 29 host plants. To be able to make this comparison, however, we had to cut pieces of fruits into an orange substrate to test odour stimuli, which is the major stimulus in the Tephritidae family^49^. While visual and tactile stimuli may influence tephritid preference, we chose to focus on olfactory stimuli which are major cues in tephritid host location^50^, and using similar oviposition devices for all tephritids. Generalist tephritids are attracted by fruits dispersed by vertebrates that are constraint by their shape and colour to attract vertebrates^40^. These fruits are mostly spherical in shape with bright colours such as the artificial device used in our study. Specialist species feeding on Cucurbitaceae also feed on smooth and round shaped fruits, and even if specialists do prefer green colour, they are also highly attracted to orange^51^. In our study specialist tephritids were indeed able to discriminate Cucurbitaceae species using only the olfactory cues supporting the importance of this cue. It would be interesting to investigate mechanisms used by specialist species to select their hosts. One could select fewer fruit species, but use the entire fruit to include the visual and tactile stimuli, creating experimental arenas that would be closer to *in natura* conditions. This would be an opportunity to look at the mechanistic differences involved in host choice between generalists and specialists.

All tested species belong to the same community on La Reunion and inhabit the same environment, but some environmental factors may modulate the relationship between female preference and larval performance. For example, in the studied community interspecific competition and host availability may affect preference-performance relationships in the field^29^. In the field, hosts of generalist species are available only few months in the year with variable abundances^29^. Generalist species may thus choose the most abundant fruit species, but not the one of highest quality. In contrast, hosts for specialists are available all year in La Réunion^29^ hence specialists can indeed choose the most suitable host. In the tephritid community host availability may reinforce the preference-performance relationship similar to the degree of specialisation. Interspecific competition generates the choice of non-optimal host for larvae. It would thus be interesting to compare the degree of specialisation of each species found in our study with the degree of specialisation in the field, taking into account environmental factors that may constrain specialisation.

Another reason why the fundamental niche in host preference may not be correlated with the optimal foraging strategy is the arrival of the insect into a new ecological context, for example in the case of biological invasions. When an insect encounters a new plant that is not optimal for larval survival, natural selection can take time to decrease the probability of females laying eggs on it or to increase the ability of larvae to survive on it^52^. Generalist species of our community have successively invaded the island over time. *Ceratitis catoirii* is the only generalist species that is native to La Réunion, while *B*. *zonata* is the most recent invader that spread through the island in 2000. We would expect a stronger relationship between preference and performance for *C*. *catoirii* than the other generalists and a stronger relationship for *D*. *demmerezi*, which is native from the Indian Ocean, compared to *Z*. *cucurbitae*, which invaded the island in 1972. The different times at which each species invaded the island may have affected the interaction between non-native species and hosts to modulate the relationship between preference and performance, where native species have a stronger relationship due to ongoing coevolution with the hosts^52^. In our study, the relationship between preference and the performance is, however, strong for both specialist species and non-existent for all generalist species. The reason could be that most host plants on La Réunion are also exotic, which means that native flies did not have a longer evolutionary history with their host plants than invasive ones. For example, among the host plants of specialists, melon originates from Africa, cucumber from Asia (like *Z*. *cucurbitae*) and pumpkin from South America^53^. In the tephritid community, the evolutionary history between fruit flies and their host plants is not very different among species due to mutual invasions.

## Methods

### Studied species and rearing conditions

Two specialist species, *D*. *demmerezi* and *Z*. *cucurbitae*, known to feed mostly on plants belonging to the cucumber family (Cucurbitaceae) and four generalist species, *C*. *catoirii*, *C*. *capitata*, *C*. *quilicii* and *B*. *zonata* ^27,30^ were used in the current study. *Ceratitis catoirii* is described as a generalist species, even though it has four hosts in the field^31^ due to interspecific competition following invasion by the three other generalist species^32^. Larvae of *C*. *catoirii*, *C*. *capitata*, *C*. *quilicii* and *B*. *zonata* were reared on an artificial diet composed of dehydrated carrot powder, brewer’s yeast, sugar, dehydrated potato, water, Nipagin/Sodium benzoate, HCl (1.65%), agar and wheat germ^54,55^ and have been in the laboratory for respectively 40, 18, 3 and 37 generations. Larvae of *D*. *demmerezi* and *Z*. *cucurbitae* were reared on zucchini for respectively 9 and 26 generations. For the experiments, naïve females were used (with no prior oviposition experience) at the age where maximum fecundity peaks (*C*. *catoirii:* 21–25 days; *C*. *capitata*: 10–15 days; *C*. *quilicii:* 14–16 days, *B*. *zonata:* 28–32 days; *D*. *demmerezi:* 13–15 days, and *Z*. *cucurbitae:* 25–30 days)^56,57^ and based on unpublished data. The different species were maintained at 25 °C, 65% RH with a 12:12 photoperiod for rearing and experiments. Details of the rearing protocol are given in Hafsi, *et al*.^35^.

### Adult preference

We evaluated the female preference by two types of oviposition experiments i.e. a choice and a no-choice experiment. Visual^58^ and tactile^59^ stimuli may be important in female preference, but olfaction is primarily used to locate hosts^60^. We, therefore, tested oviposition preference based only on olfactory cues. To homogenize visual and tactile stimuli among fruits species, an artificial oviposition device was used to compare Tephritidae female preference, as described in other studies^56,57,61^. For all experiments each fruit was chosen at the maturity stage during which the fruit would be attacked in the field. We inserted a piece of fruit, including pulp and peel, into an artificial oviposition substrate made from an orange table-tennis ball (diameter 4 cm), cut in half, and pierced with 48 evenly spaced holes. The half ball with fruit was then placed into a plastic base of suitable diameter. A source of water also was provided to the flies. Eggs laid in substrates were collected and counted after 24 hours. This substrate had already been used successfully in a previous study to assess the fecundity of Tephritidae species^56,57,61^. Before each experiment, all equipment was soaked in a detergent bath (TFD4, Dominique Dutscher SAS, Brumath, France) for 1 hour and then rinsed with running water. To verify that females were not laying eggs haphazardly (e.g. due to sensory problems or an overload of eggs), but because they discriminated the fruit, we put an empty substrate to compare the number of eggs laid in the empty *vs*. the fruit substrate. In 90% trials, no eggs were laid in the empty substrate, with a maximum of four eggs found in one trial.

To assess adult preference, we performed no-choice experiments where females were allowed to oviposit on 29 host plants belonging to 15 families that are abundant and commonly attacked by Tephritidae species on La Réunion (Table 1). In the no-choice trials, flies were presented with a single fruit at a time. Five females were put in an upside down plastic glass (h = 15 cm, bottom diameter = 9.5 cm, top diameter = 6 cm). For each Tephritidae and fruit species combination, six replicates were run. For choice experiments a smaller subset of eight fruits (Table 1) was used to evaluate whether the number of eggs laid in a single-fruit environment was indeed an adequate substitute for the preference hierarchy observed when flies had a choice between fruits. Here, 10 females were put in a cubic cage (height, length and width of 47 cm) with nine substrates in total: eight containing a piece of a different fruit and an empty one considered as a negative control. For each species, 12 replicates were run with substrates randomly placed in the cage for each replicate. The comparison of oviposition preference (estimated as number of eggs laid) between choice and non-choice experiments showed a significant positive correlation for all Tephritidae species except for two species. This supports our use of the number of eggs laid in no-choice environments as a reasonable measure of oviposition preference, similar to procedures described by others^62^. This correlation was significant for specialist species (*D*. *demmerezi*: *r* = 0.38, p-value < 0.001; *Z*. *cucurbitae*: *r* = 0.43, p-value < 0.0001) and for two generalist species (*C*. *capitata: r* = 0.29, p-value = 0.003; *B*. *zonata: r* = 0.39, p-value < 0.0001), while positive but not significant for the two others (*C*. *catoirii: r* = 0.12, p-value = 0.24; *C*. *quilicii: r* = 0.12, p-value = 0. 24) (Supplementary Fig. S2).

### Larval performance

Larval performance was assessed by measuring larval survival for the six Tephritidae species on the 29 host plants by placing one neonate larva of each species in 5 g of a diet made from 250 g of fruit pulp without peel and seed, 4 g of agar-agar and 100 ml of a solution with 0.35 g of sorbic acid potassium salt in 100 ml of distilled water for *N*. *cyanescens*, *Z*. *cucurbitae* and *D*. *demmerezi* and a solution of 4% Nipagin/sodium benzoate solution for the other species. Every 48 h during 60 days, all cups were examined and pupae collected. Larval survival was recorded as the proportion of pupae recovered from each host. For each combination of fruit and Tephritidae species, between 30 and 50 replicates were performed, except for 3 combinations where 9 (watermelon/*C*. *quilicii*), 11 (Indian almond/*C*. *quilicii*) and 11 (rose apple/*C*. *quilicii*) replicates were made due to rearing problems. Details of the experimental set-up were previously reported in the study of Hafsi, *et al*.^35^.

### Statistical analyses

We first evaluated whether our classification of the six species as specialists or generalists based on the reported number of host plant families they attack is robust for our community of Tephritidae (because some generalist species may show differences at the population-level in number of hosts attacked, i.e. level of specialisation^40,63^). We did this by calculating the alpha diversity, which represents the diversity of host plants in the preference and performance of each tephritid species, using our own oviposition and survival data. This also allowed us to move beyond the dichotomous assignment into one category or the other, providing a measure of the degree of specialisation. We calculated the alpha diversity as numbers equivalents of Shannon entropy using the VEGETARIAN package^64^. The numbers equivalents correspond to the number of equally likely elements needed to produce the given value of the diversity index. This means that the alpha host diversity of each tephritid species is essentially in the units of the number of equally common host plants^65^. For example, a fly species having 10 hosts used heterogeneously but an equivalent number of alpha diversity of 2,08 means that this fly species could be equally distributed in 2,08 plant species. To be able to calculate mean alpha diversity values and estimate variances around those means, we calculated 1,000 separate interaction matrices by randomly sampling one replicate (oviposition) or five replicates (survival) per combination of fruit/fly species. This generated an interaction matrix of 29 rows (plants) and 6 columns (fly species). We calculated the linear correlation between the alpha diversity of the adult preference and of the larval performance using the Pearson correlation index (*r*). We then used the same correlation index (*r*) to assess the linear correlation between the number of eggs laid in the choice and the no-choice experiments. Given the correlations found (see Results/Supplementary Fig. S2) hereafter the number of eggs laid under no choice conditions is used as our measure of oviposition preference.

We analysed oviposition preferences (expressed by the number of eggs) using a Poisson log-linear model (analysis of deviance with quasi-Poisson error structure to account for overdispersion) as a function of fruit species and alpha diversities (as an estimate of the degree of specialisation) and the interaction between these two variables.

Using the rate of survival of larvae as a measure of performance, we assessed the effect of the degree of specialisation on the relationship between preference and performance using a Poisson log-linear model exactly as above, but also including larval performance as a predictor variable. Finding a significant effect of the degree of specialisation, we then further examined specialists and generalists independently using two separate models. In each case, oviposition preference was analysed using a Poisson log-linear model as a function of larval survival, species and their interaction.

## Electronic supplementary material

Supplementary information
