# Supplementary material for: Diet breadth modulates preference - performance relationships in a phytophagous insect community
Source: Sci Rep. 2017 Dec 5;7:16934. doi: 10.1038/s41598-017-17231-2 (PMC5717236; doi:10.1038/s41598-017-17231-2)
Supplement: Supplementary file 1 — Supplementary information [file 41598_2017_17231_MOESM1_ESM.docx]

**Supplementary information**

Title: Diet breadth modulates preference - performance relationships in a phytophagous insect community

Authors:

Maud Charlery de la Masselière, Benoît Facon, Abir Hafsi & Pierre-François Duyck.


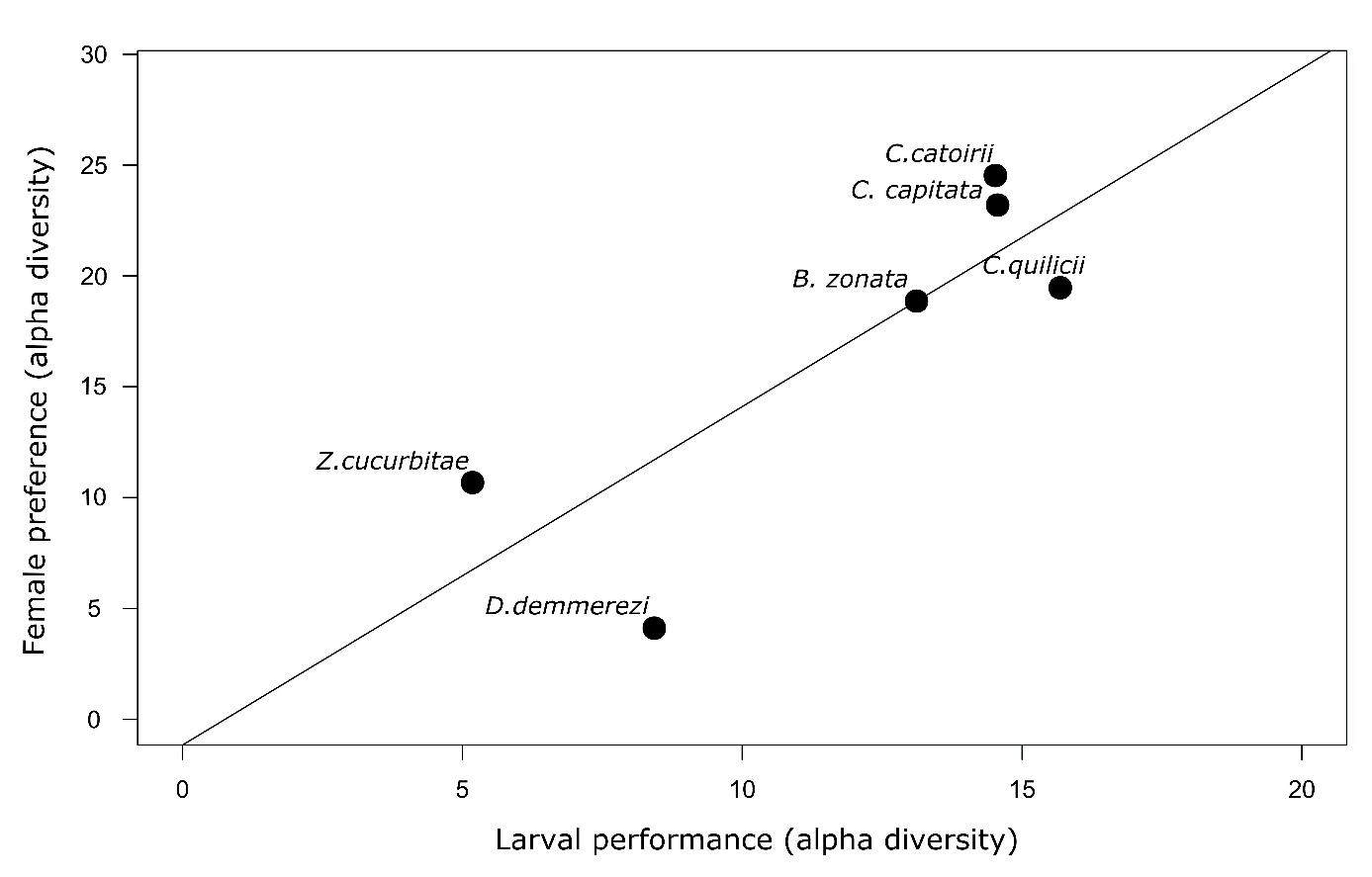


**Supplementary Figure S1.** Correlation between the alpha diversity in number equivalents of the preference and the performance of all species.


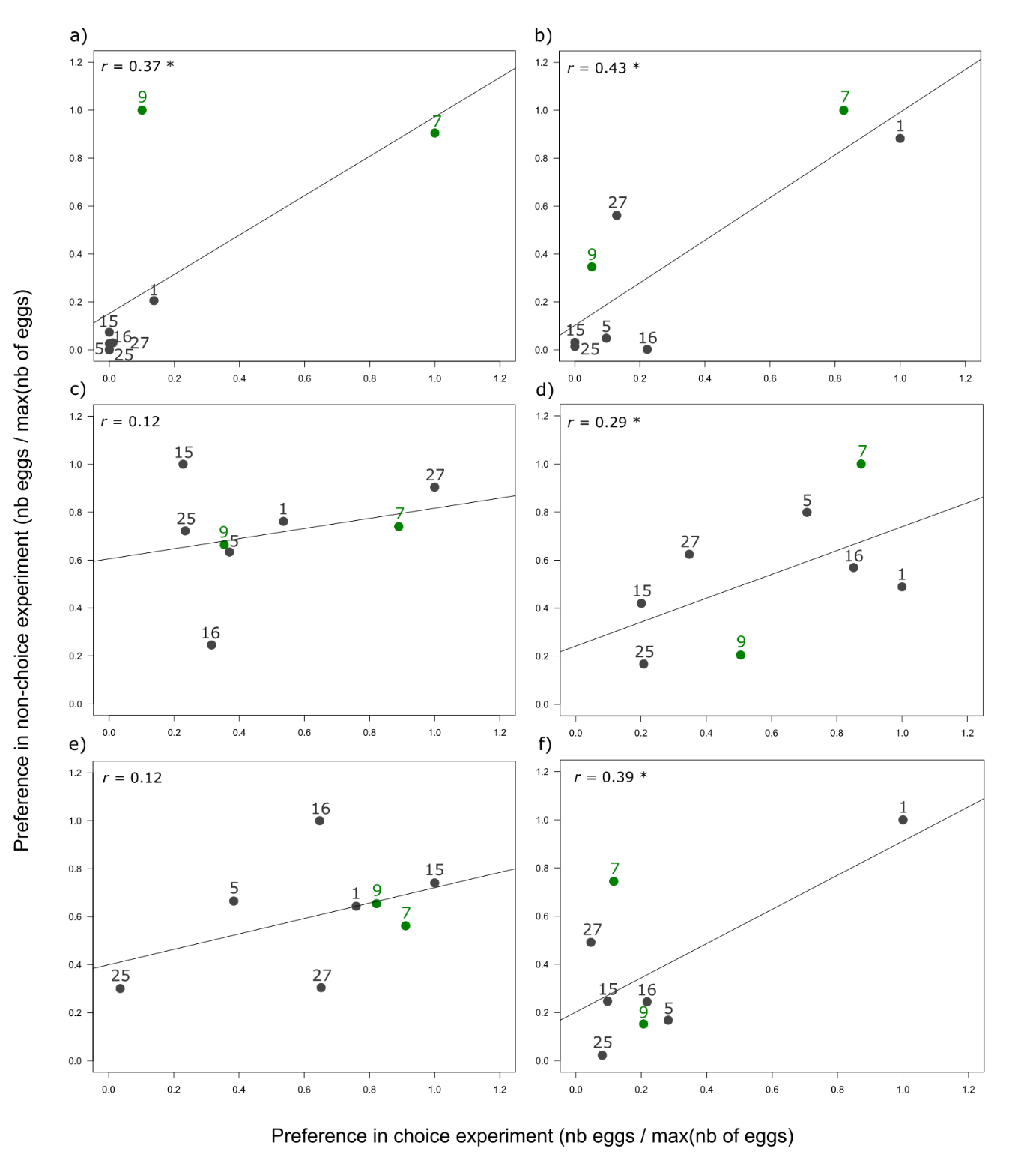


**Supplementary Figure S2.**  Relationship between preferences of females in choice and no-choice experiments for a) *D. demmerezi* b) *Z. cucurbitae* c) *C. catoirii* d) *C. capitata* e) *C. quilicii* and f) *B. zonata*. The relative preference in choice and no-choice experiments in relation to the maximum number of eggs was used. Fruits are represented by ID (Mango: 1, Indian almond: 5, Melon: 7, Pumpkin: 9, Strawberry guava: 15, Guava: 16, Chili: 25 and Tomato: 27) and dots in green represent plants of the Cucurbitaceae family.
